# High-Quality Genome Assembly of Oleaginous Red Yeast *Sporobolomyces roseus* CGMCC 2.4355

**DOI:** 10.1093/gbe/evab258

**Published:** 2021-11-22

**Authors:** Chunji Li, Ping Cheng, Yunhao Sun, Di Qin, Guohui Yu

**Affiliations:** 1 Innovative Institute for Plant Health, Zhongkai University of Agriculture and Engineering, Guangzhou, China; 2 College of Agriculture and Biology, Zhongkai University of Agriculture and Engineering, Guangzhou, China

**Keywords:** *Sporobolomyces roseus*, oleaginous red yeasts, biotechnology, genome assembly, evolutionary relationship

## Abstract

*Sporobolomyces roseus* is an important oleaginous red yeast with critical biotechnological applications and has received significant recognition as a valuable source of industrial enzymes, carotenoids, and lipids. To reveal the genetic basis and functional components underlying its biotechnological applications, a high-quality genome assembly is required. Here, we present a novel genome assembly of *S. roseus* CGMCC 2.4355 using a combination of Illunima and Oxford Nanopore technologies. The genome has an assembly size of 21.4 Mb and consists of 15 scaffolds with an N50 length of 2,126,566 bp and GC content of 49.52%. The assembly is of high integrity, comprising 95.2% complete Benchmarking Universal Single-Copy Orthologs (BUSCOs) as evaluated by a genome completeness assessment. The genome was predicted to contain 8,124 protein-coding genes, 6,890 of which were functionally annotated. We believe that the combination of our analyses and high-quality genome assembly will promote the basic development of *S. roseus* as an agent for biotechnological applications and make a significant contribution to assess the evolutionary relationship of *Sporobolomyces* species.

Significance

The type strain, *Sporobolomyces roseus* CGMCC 2.4355, is an oleaginous red yeast with significant industrial potential for the production of enzymes, carotenoids, and lipids. Nevertheless, despite its potential biotechnological importance, only a limited genome sequence is currently available for this species. This study was able to produce the first high-quality genome assembly for *S. roseus* CGMCC 2.4355, providing a critical tool for identifying candidate genes underlying the biotechnological potential of this microorganism.

## Introduction

*Sporobolomyces roseus* is a well-studied member of the *Sporobolomyces* genus and was first reported in 1924 (Hamamoto et al. 2011). This species is of significant biotechnological importance and is commonly used as a promising platform for the industrial production of various valuable metabolites (Buzzini et al. 2007). The most apparent advantage of this species is its ability to produce valuable compounds using low-cost waste material substrates, thereby greatly enhancing the economic benefits of bioprocesses (Marova et al. 2012).

One of the major characteristics of *S. roseus* is its production of aspartic protease (Białkowska et al. 2018), urease (Jahns 1995), and phenylalanine/tyrosine ammonia lyase (PAL/TAL) (Camm and Towers 1969). These enzymes could potentially be used in several industrial sectors given their health-promoting properties, especially the PAL proteins. The orange to salmon-pink color of *S. roseus* was found to be the result of an accumulation of carotenoids such as β-carotene, torulene, and torularhodin (Davoli and Weber 2002). Previous studies have shown that β-carotene supplementation can improve vitamin A uptake and antioxidant production, reducing the incidence of several chronic diseases (Bohn et al. 2019). Although both torulene and torularhodin are less common carotenoids, the limited number of studies describing their effects suggest that both compounds exhibit stronger antioxidant properties than those of β-carotene (Kot et al. 2018), with one previous study even linking them to the prevention of prostate cancer (Du et al. 2016). In addition, torularhodin ingestion significantly decreases ethanol-induced alcoholic liver disease and hepatic oxidative damage (Li et al. 2019). Torularhodin also possesses strong antimicrobial properties, making it a candidate for developing novel commercial antibiotics (Ungureanu et al. 2016). Moreover, *S. roseus* can act as an efficient biocatalyst for synthesizing diverse lipids, including vaccenic, linoleic, palmitic, and stearic acids (Ananda and Vadlani 2010), which are all raw materials for biodiesel production and important sources of unsaturated fatty acids (Waché et al. 2006). Therefore, *S. roseus* is widely regarded as a versatile yeast with significant biotechnological potential.

Although several studies have described the industrial application of *S. roseus*, its high-resolution genome is still lacking. Considering the necessity of whole genome sequencing in identifying the underlying molecular mechanisms responsible for its biotechnological applications, we decided to complete a high-quality genome assembly for *S. roseus* type strain CGMCC 2.4355. This genome assembly will facilitate the identification of candidate genes for the biotechnological application of *S. roseus* and aid in developing comparative genomics studies designed to evaluate the evolutionary dynamics of *Sporobolomyces* species.

## Results and Discussion

### Genome Sequencing, Assembly, and Completeness Assessment

**Fig. 1.**
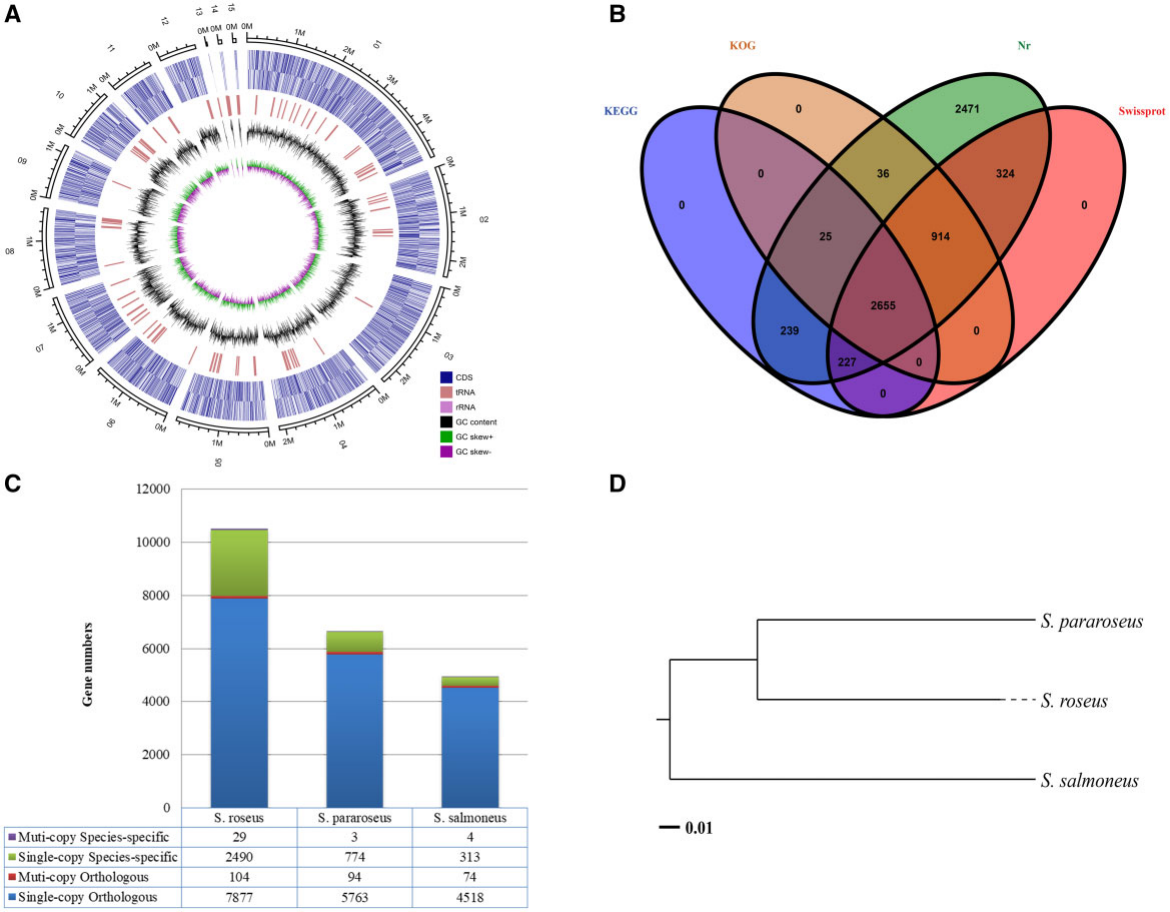
(*A*) Genomic landscape of *S. roseus* CGMCC 2.4355. (*B*) Venn diagram illustrating shared and unique genes annotated in the NR, SwissProt, KOG, and KEGG databases. (*C*) Distribution of orthologous and species-specific genes within three sequenced *Sporobolomyces* species. (*D*) Phylogenetic tree of three sequenced *Sporobolomyces* species was constructed based on aligned orthologs with Neighbor-Joining method (bootstrap: 1,000 replicates).

Our sequencing produced 4.57 Gb of Oxford Nanopore long reads (∼200×) and 1.42 Gb (∼70×) of Illumina short reads, which were then combined and used to produce a novel genome assembly for *S. roseus* type strain CGMCC 2.4355. These Oxford Nanopore reads were corrected using the Illumina reads and the FMLRC 1.0.0 program. These were subsequently used to produce an assembled genome consisting of 15 scaffolds (fig. 1*A*) with an N50 length of 2,126,566 bp and maximum and minimum scaffold lengths of 4,742,556 and 23,714 bp, respectively. This genome was shown to have a GC content of 49.52% and was approximately 21.4 Mb in size (table 1). In addition, we identified a total of 1,271 (95.2%) complete BUSCOs, which completely covered the assembled genome when evaluated using BUSCO alignment (supplementary table S1, Supplementary Material online), indicating that this assembled genome was of high quality and largely complete.

**Table 1.** Summary of Assembly Statistics

Assembly	Size (bp)	22,396,975
	Number of scaffolds	15
	Scaffold N50 (bp)	2,126,566
	Scaffold N90 (bp)	1,104,580
	Longest scaffold (bp)	4,742,556
	Shortest scaffold (bp)	23,714
	GC content (%)	49.52
BUSCO	Complete and single-copy BUSCOs	1,266
	Complete and duplicated BUSCOs	5
	Fragmented BUSCOs	22
	Missing BUSCOs	42
	Total BUSCOs searched	1,335
Repetitive elements	SINEs (bp)	2,206
	LINEs (bp)	6,840
	LTR (bp)	930
	DNA transposons (bp)	2,446
	Total (bp)	12,422
Annotation	Predicted genes	8,124
	Functional-annotated genes	6,890
	Mean gene length (bp)	2,391.07
	Exons/gene	6.98
	Introns/gene	5.98
	Exon ratio (%)	67.96
	Intron ratio (%)	18.77
	Mean exon length (bp)	268.38
	Mean intron length (bp)	86.54

### Functional Annotation

Protein prediction identified 8,124 protein-coding genes within the *S. roseus* CGMCC 2.4355 genome with a total length of 13,548,786 bp, maximum and minimum lengths of 16,041 and 99 bp, respectively, and a mean GC content of 50.51%. Thereafter, BlastP evaluation annotated a total of 6,890 (84.81%) of these genes (e-value <1e^−5^) using sequence homology and the NCBI Nr database. Moreover, 4,119 (50.7%), 3,629 (44.67%), and 3,145 (38.71%) of the genes were annotated using the SwissProt, Clusters of Eukaryotic Orthologous Groups (KOG), and Kyoto Encyclopedia of Genes and Genomes (KEGG) databases (fig. 1*B*), respectively. KEGG mapping identified several candidates explaining the biotechnological potential of *S. roseus* CGMCC 2.4355. Briefly, these candidate gene inventories were as follows: 1) synthesis of industrial enzymes, including genes encoding lipases, aspartyl proteases, ureases, and PAL; 2) synthesis of carotenoids, including geranylgeranyl diphosphate synthase (*crtE*), phytoene synthase/lycopene beta cyclase (*crtYB*), phytoene dehydrogenase (*crtI*), carotenoid cleavage dioxygenase (*CCD*), and carotenoid ester lipase precursor; 3) lipid metabolism, including genes encoding acyl CoA oxidase (ACOX3), phospholipid diacylglycerol acyltransferase (PDAT), and acetyl CoA carboxylase (ACACa).

### Phylogenetic Analysis

The evolutionary relationship of the *Sporobolomyces* species were calculated using pairwise comparisons among the genomes of *S. roseus* CGMCC 2.4355, *S. pararoseus* CGMCC 2.5280, and *S. salmoneus* CBS 6832. These evaluations identified 9,254 gene families and 3,407 one-to-one orthologs between these species. Of these, we found 2,519 (2,577 genes), 777 (780 genes), and 313 (320 genes) species-specific gene families in *S. roseus* CGMCC 2.4355, *S. pararoseus* CGMCC 2.5280, and *S. salmoneus* CBS 6832 (fig. 1*C*), respectively. Phylogenetic analysis indicated that *S. roseus* has a closer evolutionary relationship with *S. pararoseus* than that of *S. salmoneus* (fig. 1*D*).

## Conclusions

Our study is the first to report a high-quality genome assembly for the biotechnologically important oleaginous red yeast *S. roseus* CGMCC 2.4355. This assembly facilitated the identification of several candidate protein-encoding genes involved in the production of industrially important enzymes, carotenoids, and lipids which have been duly annotated in this genome. Taken together, this work represents a cornerstone study of *S. roseus* as a biocatalyst for developing genetically engineered compounds, facilitating comparative genomics studies of evolutionary dynamics within the *Sporobolomyces* genus.

## Materials and Methods

### Yeast Strain and Growth Conditions

*S. roseus* CGMCC 2.4355 was obtained from the China General Microbiological Culture Collection Center (Beijing, China). It was found to produce three kinds of carotenoids (β-carotene: 630.45 µg/g_dw_; torulene: 307.25 µg/g_dw_; torularhodin: 51.35 µg/g_dw_) and 839 kinds of lipids (supplementary table S2, Supplementary Material online). *Sporobolomyces roseus* CGMCC 2.4355 cells were prepared in 500 ml Erlenmeyer flasks containing 150 ml of yeast extract–peptone–dextrose medium (yeast extract: 10 g/l, peptone: 20 g/l, dextrose: 20 g/l). Cells were harvested by centrifugation and immediately frozen in liquid nitrogen for genomic DNA and total RNA extraction.

### DNA Extraction and Genome Sequencing

Genomic DNA extraction was performed using a Genomic DNA Extraction Kit (Invitrogen, Waltham, MA) according to the manufacturer’s instructions. The extracted DNA was then used for whole genome sequencing using the Nanopore long-read sequencing platform (PromethION; Oxford Nanopore Technologies, Oxford, UK) and the Illumina short-read sequencing platform (Novaseq 6000; Illumina, San Diego, CA) at Gene Denovo Biotechnology Co., Ltd. (Guangzhou, China).

### Genome Assembly

Raw reads from the Illumina sequencing were first used to correct the Nanopore raw reads using the FMLRC program (version 1.0.0) at -k 21 and -K 59. These corrected Nanopore reads were then used to complete a de novo assembly in wtdbg2 (version 2.4) at -p 21, -k 0, -AS 4, -K 0.05, and -s 0.5. Thereafter, the Illumina raw reads were used to correct the primary genome assembly and determine the final genome sequence using Pilon (version 1.23) at –K 47—mindepth, 0.1 –mingap 10, and –flank 10. Completeness of the genome assembly was evaluated using the BUSCO program (version 3.0.1) and its “basidiomycota_odb9” data set.

### RNA Extraction and Transcriptome Sequencing

Total RNA extraction was performed using the Trizol Reagent Kit (Takara Bio Inc, Shiga, Japan) according to the manufacturer’s protocol, and high-quality total RNA samples were then used to prepare cDNA libraries for transcriptome sequencing on the Illumina short-read sequencing platform (Novaseq 6000; Illumina, San Diego, CA) at Gene Denovo Biotechnology Co., Ltd. (Guangzhou, China).

### Gene Prediction and Functional Annotation

Open reading frames were predicted using the GeneMark-ES program (version 4.35) set to default parameters. Next, raw reads from the transcriptome sequencing were mapped against the assembled genome to predict gene models using the GeneMark-ET program set to default parameters. Subsequently, these predicted genes were annotated by aligning them with the deposited gene sequences in several diverse protein databases, including the NCBI nonredundant protein (Nr), SwissProt, KEGG, and KOG databases.

### Phylogenetic Analysis

The annotated gene sequences from *S. pararoseus* CGMCC 2.5280 and *S. salmoneus* CBS 6832 were downloaded from the NCBI Genome database and aligned with the *S. roseus* CGMCC 2.4355 assembly. Genome alignments were conducted using an all-against-all comparison in MUMmer 3 (version 3.2.2) set to default parameters. The OrthoMCL (version 2.0) program was then used to identify core orthologs with a cut-off value of 1 × 10 ^−7^. Sequence alignments of orthologs were performed using the MUSCLE program (version 3.8.31) with default parameters (Edgar 2004). Phylogenetic tree was constructed using the MEGA 7.0 software based on aligned orthologs with Neighbor-Joining method.

## Supplementary Material

Supplementary data are available at *Genome Biology and Evolution* online.

## Supplementary Material

evab258_Supplementary_Data
